# Evaluation of neonatal and maternal morbidity in mothers with gestational diabetes: a population-based study

**DOI:** 10.1186/s12884-018-2005-9

**Published:** 2018-09-10

**Authors:** Grzegorz Domanski, Anja Erika Lange, Till Ittermann, Heike Allenberg, Robert Andreas Spoo, Marek Zygmunt, Matthias Heckmann

**Affiliations:** 1 Department of Neonatology and Pediatric Intensive Care, University Medicine Greifswald, Ferdinand-Sauerbruch-Strasse, 17487 Greifswald, Germany; 2 Institute for Community Medicine, Div. SHIP – Clinical Epidemiological Research, University Medicine Greifswald, Walter Rathenau Str. 48, 17475 Greifswald, Germany; 3 Department of Gynecology and Obstetrics, University Medicine Greifswald, Ferdinand-Sauerbruch-Str, 17487 Greifswald, Germany

**Keywords:** Gestational diabetes mellitus, Pregnancy, Survey of neonates in Pomerania, SNiP, Risk factors

## Abstract

**Background:**

Gestational diabetes mellitus (GDM) is the most frequent complication during pregnancy. Untreated GDM is a severe threat to maternal and neonatal health. Based on recent evidence, up to 15% of all pregnancies may be affected by GDM. We hypothesized that in a rural birth cohort, higher maternal BMI and adverse socioeconomic conditions would promote GDM, which in turn would lead to adverse effects on pregnancy outcomes.

**Methods:**

The current study is a part of a population-based cohort study examining the health and socioeconomic information from 5801 mothers and their children. The study, titled the Survey of Neonates in Pomerania (SNiP), was based in northeastern Pomerania, Germany (2002–2008).

**Results:**

The cumulative incidence of GDM was 5.1%. Multiple logistic regression revealed prepregnancy overweight (OR 1.84 (95% CI 1.27–2.68)), prepregnancy obesity (OR 3.67 (2.48–5.44)) and maternal age (OR 1.06 (1.03–1.08)) as risk factors for GDM (*p* = 0.001). Alcohol use during pregnancy (OR 0.61 (0.41–0.90), a higher monthly income (OR 0.62 (0.46–0.83)), and the highest level of education (OR 0.44 (0.46–0.83)) decreased the risk of GDM.

Newborns of GDM mothers had an increased risk of hypoglycaemia (OR 11.71 (7.49–18.30)) or macrosomia (OR 2.43 (1.41–4.18)) and were more often delivered by primary (OR 1.76 (1.21–2.60)) or secondary C-section (OR 2.00 (1.35–2.97)). Moreover, they were born 0.78 weeks (95% CI -1.09 – -0.48 weeks) earlier than infants of mothers without diabetes, resulting in higher percentage of late preterm infants with a gestational age of 32–36 weeks (11.1% vs. 6.96%).

**Conclusions:**

Age and BMI before pregnancy were the predominant mediators of the increased risk of GDM, whereas a higher income and educational level were protective. GDM affected relevant perinatal and neonatal outcomes based on its association with an increased risk of delivery by C-section, preterm birth, macrosomia at birth and neonatal hypoglycaemia.

## Background

Gestational diabetes mellitus (GDM) is one of the most frequent complications during pregnancy. According to official figures, its prevalence in Germany more than doubled between 2002 and 2010, reaching 3.7% in 2010 [1, 2]. The worldwide prevalence of GDM varies between 1 and 22%, depending on the genetic background of the population under study, employed diagnostic methods and environmental factors [3, 4]. The recent report published by International Diabetes Federation (IDF) states that one in seven births might be affected by GDM [5]. At the national level, similar figures have recently been published by Melchior and colleagues, who analysed pregnancies in Germany in the years 2014 and 2015 [6].

Untreated GDM is a severe threat to maternal and neonatal health [1, 7, 8]. For example, the maternal risk for pre-eclampsia and/or eclampsia increases 8-fold. Likewise, the increased risk of pregnancy-associated hypertension and the incidence of childbirth injuries are caused by adjustment anomalies, macrosomia of the neonate and any necessary C-section [7]. A significant number of women with GDM, up to 50%, develop type 2 diabetes later in life [9, 10]. The offspring of mothers with GDM experience elevated blood glucose levels and changes in amino acid and lipid profiles, which stimulate the secretion of insulin and growth factors [11]. As result, these children are at a 6-fold higher risk of type 2 diabetes in childhood and adolescence [12, 13].

An increasing number of publications reporting results from childbirth cohort studies indicate that not only genetic background but also sociodemographic factors and expectant mothers’ lifestyles influence the incidence of GDM [14–18]. According to results from the Generation R Study [14], low maternal educational level promoted the development of GDM. An Italian study from Turin found that mothers with low socioeconomic position (SEP), a composite index assessing educational level and employment, were at a higher risk of developing GDM [15]. However, other risk factors, such as alcohol use, smoking, unhealthy diet, and stress, may play a role in the development of GDM [16–18].

The present study is part of the population-based birth cohort study “Survey of Neonates in Pomerania (SNiP)”. The SNiP delivers comprehensive information about the health and socioeconomic status of > 95% of the newborns in a geographically defined study region. While other cohort-based studies, such as LIFE in Leipzig [19] or Generation R [20], investigate urban populations with highly inhomogeneous ethnic compositions, the SNiP was conducted in a rural area on a population with high prevalence of obesity and low socioeconomic status compared to the populations in other regions of Germany [19, 21, 22].

Our aim was to investigate how maternal health status and family socioeconomic status (educational level and income) was associated with the risk of GDM. Furthermore, the effect of GDM on pregnancy and the offspring was analysed. Our hypothesis was that higher maternal BMI and adverse socioeconomic conditions are associated with a higher prevalence of GDM, which, in turn, leads to adverse effects on pregnancy outcomes in a rural birth cohort.

## Methods

### Study design

Details of the SNiP have been reported by Ebner and colleagues [23]. The SNiP was conducted from 2002 to 2008 in the region of Pomerania in northeastern Germany. Personal data, medical records data (149 variables), personal interview data (84 variables), and data from a self-administered questionnaire concerning socioeconomic background (40 variables) were collected and recorded from each participating mother and child. From all nonparticipants, excluded individuals and nonresponders, a minimum dataset was compiled comprising data on the health status of women and their newborns but lacking detailed information about environmental parameters.

### Population

**Fig. 1:**
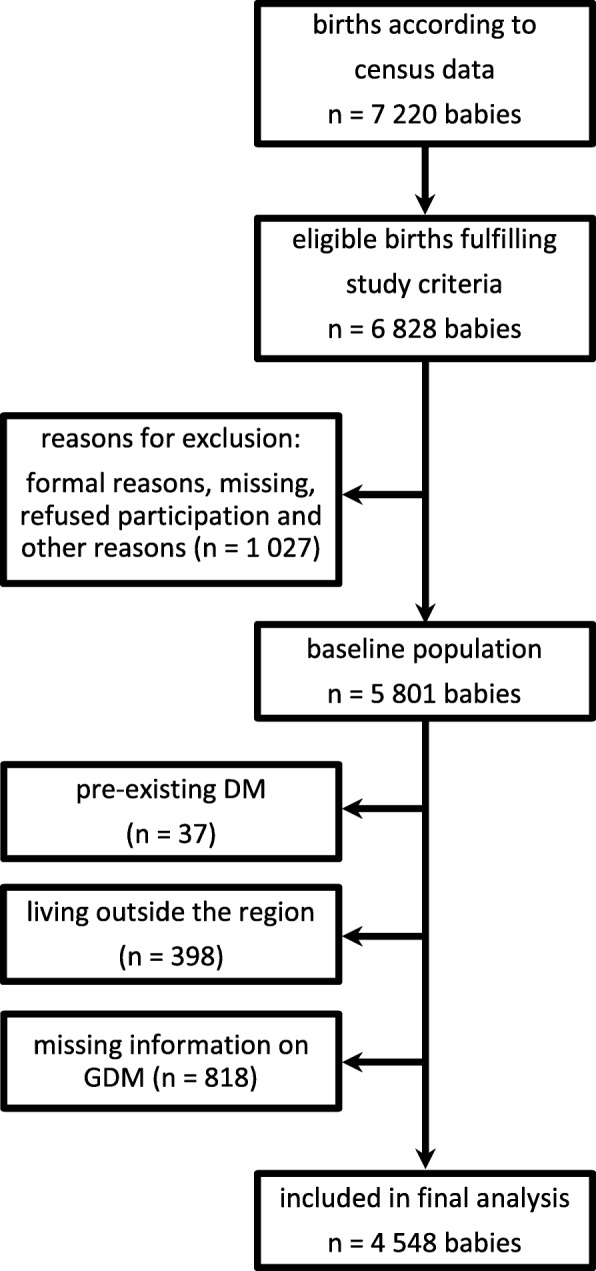
Selection tree and sample description for the baseline population and the analysed subpopulation

The selection tree and sample description for the baseline population and analysed subpopulation is shown in Fig. 1.

### Educational level

The stratification pattern was chosen following Lange et al. [24], with some modifications. Due to a low number of cases, the two lowest educational levels (“did not earn a school diploma” and “still at school”) were combined with the level “5 years of secondary school”. This pool of cases was referred to as having a low educational level. Persons with 6 years of secondary school (Realschulabschluss) were included in the second level, referred to as the middle educational level. The third level included persons with 8 years of secondary school (Fachhochschulreife or Abitur) and was referred to as the mid-high educational level. The highest educational level was assigned to persons who have graduated and is referred to as a high educational level.

### Definitions of smoking and alcohol use

In this paper, we did not analyse the dose effect of tobacco and alcohol consumption on pregnancy outcomes. Therefore, we did not differentiate the cohort by the amount of alcohol consumed or tobacco smoked. Instead, we used a simple dichotomous classification: “smoker/nonsmoker” and “drinker/nondrinker”. A woman was classified as a smoker if she declared that she smoked during the last four weeks before delivery. Similarly, a woman was classified into the group of drinkers if she continued to drink alcohol during pregnancy, irrespective of the amount and time period of consumption.

### Criteria for inclusion as a mother with GDM

During the study period, there was no obligatory screening for GDM in Germany using a 75-g oral glucose tolerance test (75-g oGTT). According to national German maternity policy guidelines (MPG, “Mutterschafts-Richtlinien”), which were valid during the data collection [25], the midstream urine samples of all pregnant women were tested for the presence of glucose. The MPG states that the first test should be conducted “as soon as possible after finding out about the pregnancy”. In our study cohort, the first test took place after the 10th week of pregnancy (median value). The test was repeated every four weeks. Women having suspicious results were referred to the hospital, where a 75-g oGTT was administered after the women completed eight hours of fasting [1]. If at least one of the following values was exceeded, a woman was classified as having GDM:

- Fasting state: ≥ 5.1 mmol/l (92 mg/dl)
- After 1 h: ≥ 10.0 mmol/l (180 mg/dl)
- After 2 h: ≥ 8.5 mmol/l (153 mg/dl)

Only cases with confirmed GDM (ICD-10 code O24.4) were included in the current analysis.

### Definition of foetal macrosomia

For the purposes of this study, macrosomia was defined as a birth weight greater than the 97th percentile adjusted for gestational age, with cutoff values according to Voigt et al. [26].

### Diagnosis of neonatal hypoglycaemia

Neonatal hypoglycaemia was diagnosed using biochemical parameters according to national guidelines [27]. In Germany, a plasma glucose limit of 45 mg/ml (2.5 mmol/l) within the first 24 h after birth was used to diagnose neonatal hypoglycaemia. It was a routine policy in the study region to screen all babies of mothers with GDM for hypoglycaemia.

### Conditions for admission to neonatal care

All babies of mothers with GDM were closely monitored biochemically by the measurement of blood glucose levels and clinically by screening for symptoms of hypoglycaemia according to the German guideline [27]. The level of monitoring depends on the severity of symptoms. According to the institutional policy and the German guideline, the personnel attach great importance to maintaining mother-child contact even in the case of pathology. Babies and their mothers were left at the maternity ward as long as the conditions allowed such a situation. The national guideline clearly defines when the neonate should be transferred to the neonatal ward, particularly when intravenous administration of glucose was necessary or in case of severe symptoms. For the purposes of the study, “admission to neonatal care” included both neonatal intensive care and special care with respect to the newborn’s condition and needs.

### Data assessment

All mothers included in this analysis provided written informed consent to participate in the study, which was approved by the Ethics Committee of Ernst Moritz Arndt University, Greifswald. Data were collected in standardized 5- to 10-min interviews. Parents also completed a questionnaire during their stay on the ward and returned it to the medical staff before discharge. This questionnaire included questions about the parents’ social background and lifestyle. Data on the gestational period and from any preventive examinations were acquired using the mothers’ medical files and maternity cards. The collected data were anonymized and stored in a Microsoft® Access® database (Microsoft Corporation, Redmond, WA, USA).

### Potential mediators and confounders

The level of maternal education does not affect the risk of GDM directly but may act as a proximal risk factor (mediator). We have considered the following factors as potential mediators in the pathway between maternal education and GDM: smoking and alcohol consumption during pregnancy and prepregnancy body mass index. These variables were assessed by the self-administered questionnaire.

Some other risk factors cannot be considered indisputable mediators, and we treated them as potential confounders: maternal age, weight gain during pregnancy, available monthly income (equivalent income) and parity. Ethnicity is another potential confounder [14, 16, 28]; however, this factor could not be analysed because less than 2% of the population were not Caucasian.

### Definition of monthly available equivalent income

As the needs of a household grow with each additional member, this growth does not happen in a proportional manner, due to economies of scale. The need for housing space, electricity, and other essentials is not three times as high for a household with three members than for a single person. To account for this phenomenon, we have used equivalence scales, based on the OECD-modified scale [29]. The available monthly equivalent income was calculated according to the following formula:

Total income = the household’s income divided by the number of members in the household.

Moreover, each member receives the following weight:

- the first adult of the household receives a weight of 1
- other individuals older than 13 years old receive a weight of 0.5
- children receive a weight of 0.3 (0- to 13-year-old individuals are defined as children)

### Statistical analyses

All data were stored using a Microsoft Access 2002 (Microsoft Corporation, Redmond, WA, USA) database.

Continuous data are reported as the medians with the 25th and 75th percentiles; categorical data are expressed as the absolute numbers and percentages. Associations of mothers’ potential risk factors, such as age, education, equivalent income, body mass index before pregnancy and smoking and alcohol consumption during pregnancy, with the development of GDM were analysed by logistic regression models adjusted for confounders. Associations between GDM during pregnancy and outcomes of the child, such as birth weight, gestational age, hypoglycaemia, admission to the neonatal care unit, mode of delivery, respiratory distress, and mother’s vaginal infections, were analysed by linear, logistic and multinomial logistic regressions adjusted for confounders. The respective confounders used in the multivariate analyses are mentioned in the legends of Table 2 and Table 4. In all analyses, *p* < 0.05 was considered statistically significant. All *p*-values were calculated using two-tailed tests. All analyses were carried out with Stata 14.1 (Stata Corporation, College Station, TX, USA).

## Results

### General characteristics of the cohort

The characteristics of the included pregnant women and neonates are shown in Table 1 (continuous and categorical variables). The cumulative incidence of gestational diabetes mellitus (GDM) was 5.1% (*n* = 232 out of 4548). For women with and without GDM, the maternal age at birth was 29 years and 27 years (median, *p* < 0.001), the BMI before pregnancy was 24.9 and 22.3 (median, p < 0.001), and the gestational weight gain was 13 kg and 15 kg (median, *p* = 0.018). There was no significant difference in monthly equivalent income between the two groups (*p* = 0.407). In total, 46.4% (*n* = 2106) of all women but only 37.1% of mothers with GDM were nulliparous (*p* = 0.024; Table 1).

**Table 1:** Characteristics of the study population. Continuous and categorical variables were stratified by the prevalence of GDM (univariate analysis)

Variable	Total	Without GDM	With GDM^a^	*p*-value^b^
*Continuous variables*				
Maternal age, years (n)	27 (4542)	27 (4317)	29 (225)	*p* < 0.001
BMI^c^ before pregnancy, kg/m^2^ (n)	22.5 (4010)	22.3 (3814)	24.9 (196)	*p* < 0.001
GWG^d^, kg (n)	15 (3984)	15 (3789)	13 (195)	*p* = 0.018
Income, € (n)	1060 (2497)	1060 (2361)	1060 (136)	*p* = 0.407
*Categorical variables*				
Nulliparous, n (%)	2106 (46.4)	2023 (46.8)	83 (37.1)	*p* = 0.024
Current smoker (*n* = 3908)	732 (18.7)	702 (18.9)	30 (16.0)	*p* = 0.318
Alcohol use during pregnancy (*n* = 4008)	968 (24.2)	935 (24.5)	33 (17.0)	*p* = 0.017
BMI before pregnancy (*n* = 4010)				
Underweight (< 19), n (%)	428 (10.7)	419 (11.0)	9 (4.59)	*p* < 0.001
Normal weight (19–24.99), n (%)	2477 (61.8)	2386 (62.6)	91 (46.4)	
Overweight (25–29.99), n (%)	719 (17.9)	671 (17.6)	48 (24.5)	
Obese (> = 30), n (%)	386 (9.63)	338 (8.86)	48 (24.5)	
Education level (*n* = 3955)				
Low, n (%)	600 (15.2)	569 (15.1)	31 (15.8)	*p* = 0.845
Middle, n (%)	2052 (51.9)	1947 (51.8)	105 (53.6)	
Mid-high, n (%)	735 (18.6)	699 (18.6)	36 (18.4)	
High, n (%)	568 (14.4)	544 (14.5)	24 (12.2)	
Positive vaginal swab test (*n* = 3886)	594 (15.3)	543 (14.8)	51 (24.8)	*p* < 0.001
Preeclampsia (*n* = 4548)	108 (2.37)	103 (2.38)	5 (2.22)	*p* = 0.878

Data are expressed as medians and absolute numbers (in parentheses) for continuous variables. Categorical variables are presented as absolute numbers and percentages (in parentheses); ^a^*GDM* gestational diabetes mellitus, ^b^Wilcoxon test for continuous and two-tailed χ2 test for categorical variables, ^c^*BMI* body mass index, ^d^*GWG* gestational weight gain

A total of 86% (*n* = 3908) of women reported on their smoking behaviour, and 88% (*n* = 4008) reported on alcohol consumption before and during the pregnancy. Almost one in six women (*n* = 732, 18.7%) continued to smoke after the pregnancy was known, without significant differences between pregnant women with and without GDM (*p* = 0.318). Nearly a quarter of pregnant women (*n* = 968, 24.2%) did not stop drinking alcohol, but fewer women with GDM than women without GDM continued to drink (17.0% versus 24.5%, *p* = 0.017).

Mothers with GDM were more often overweight (24.5% versus 17.6%) or obese (24.5% versus 8.86%) but less frequently underweight (4.59% versus 11.0%) or of normal weight (46.4% versus 62.6%). A total of 24.8% of pregnant women with GDM received positive result on the swab test, compared to 14.8% of women without GDM (*p* < 0.001). There was no significant difference by univariate analysis between GDM diagnosis and mothers’ educational level (*p* = 0.845) or between the occurrence of preeclampsia and GDM diagnosis (*p* = 0.878).

### Influence of risk factors on GDM

In the next step, we calculated a multiple logistic regression to evaluate the association between risk factors and GDM (Table 2). Compared to participants of normal weight (BMI between 19 and 24.99), those who were overweight at prepregnancy had almost double the risk of GDM (OR 1.84, 95% CI 1.27–2.68, *p* = 0.001). The risk of developing GDM in women who were obese before pregnancy was more than threefold higher than that in the group of normal weight (OR 3.67, 95% CI 2.48–5.44, *p* < 0.001). Maternal age was associated with the risk of GDM. The risk increased by 6% for each additional year of mothers’ age (OR 1.06, 95% CI 1.03–1.08, p < 0.001). Alcohol use during pregnancy (OR 0.61, 95% CI 0.41–0.90, *p* = 0.01) decreased the risk of having GDM. We did not observe that gestational weight gain (OR 0.99, 95% CI 0.97–1.01, *p* = 0.48) or parity affected the risk of GDM.

**Table 2:** Associations between potential risk factors and gestational diabetes using multiple logistic regression

	OR^a^ (95% CI^b^)	*p*-value^d^
Maternal age	1.06 (1.03–1.08)	*p* < 0.001
Smoking	0.79 (0.51–1.23)	*p* = 0.29
Alcohol use	0.61 (0.41–0.90)	*p* = 0.014
Prepregnancy BMI^c^		
Underweight (< 19)	0.68 (0.34–1.36)	*p* = 0.27
Normal weight (19–24.99)	Reference	
Overweight (25–29.99)	1.84 (1.27–2.68)	*p* = 0.001
Obese (> = 30)	3.67 (2.48–5.44)	*p* < 0.001
Gestational weight gain	0.99 (0.97–1.01)	*p* = 0.48
Parity		
First child	Reference	
Second child	1.11 (0.78–1.59)	*p* = 0.55
Third child	1.28 (0.82–2.02)	*p* = 0.28
Forth child and more	0.92 (0.53–1.61)	*p* = 0.77
Education level		
Low	Reference	
Middle	0.73 (0.46–1.16)	*p* = 0.19
Mid-high	0.65 (0.38–1.13)	*p* = 0.13
High	0.44 (0.23–0.83)	*p* = 0.01
Income; thousand €	0.62 (0.46–0.83)	*p* = 0.001

^a^*OR* odds ratio, ^b^*CI* confidence interval, ^c^*BMI* body mass index, ^d^logistic regression adjusted for confounders (mother’s age, alcohol use and prepregnancy BMI)

The multiple logistic regression (Table 2) showed that a higher monthly was associated with reduced risk of GDM (OR 0.62, 95% CI 0.46–0.83, p = 0.001). The risk of having GDM decreased by 38% per additional one thousand euros of available income.

With regard to mothers’ education, only the highest level of education decreased the risk of GDM (OR 0.44, 95% CI 0.46–0.83, p = 0.01). Neither the middle nor the mid-high level of education significantly influenced the risk of GDM (*p* = 0.19 and *p* = 0.13, respectively).

Finally, we calculated a prediction model to evaluate the discriminative power of all variables with *p* < 0.1 (maternal age, prepregnancy BMI, alcohol use during pregnancy, mother’s educational status and income; see Table 2) on the occurrence of GDM. After backward elimination, the final model demonstrated a modest fit (AUC = 0.661, 95% CI 0.621–0.702) and included the following variables: maternal age, BMI between 25 and 29.9, BMI greater than or equal to 30, and alcohol use during pregnancy.

### Influence of GDM on neonatal outcomes

Fewer neonates of women with diabetes had a normal weight (75.1% versus 80.0%) or were underweight (4.89% versus 9.46%, *p* < 0.001) than neonates of women without diabetes (Table 3). In contrast, these neonates were more often overweight (20.0% versus 10.6%, *p* < 0.001) or had macrosomia (8.00% versus 3.40%, p < 0.001).

**Table 3:** Neonatal outcomes stratified by the prevalence of GDM (univariate analysis)

Variable	Total	Without GDM	With GDM^a^	*p*-value^d^
Birth weight (*n* = 4548)				
Normal weight	3626 (79.7)	3457 (80.0)	169 (75.1)	*p* < 0.001
Underweight (SGA,^b^ < 10th percentiles)	420 (9.23)	409 (9.46)	11 (4.89)	
Overweight (LGA,^c^ > 90th percentiles)	502 (11.0)	457 (10.6)	45 (20.0)	
Macrosomia, > 97th percentile (*n* = 4548)	165 (3.63)	147 (3.40)	18 (8.00)	*p* < 0.001
Gestational age (*n* = 4548)				
< 32 weeks	75 (1.65)	73 (1.69)	2 (0.89)	*p* = 0.031
32–36 weeks	326 (7.17)	301 (6.96)	25 (11.1)	
37–41 weeks	4093 (90.0)	3895 (90.1)	198 (88.0)	
> 41 weeks	54 (1.19)	54 (1.25)	0 (0.00)	
Mode of delivery (*n* = 4534)				
Spontaneous	3162 (69.7)	3037 (70.3)	125 (55.8)	*p* < 0.001
Primary section	664 (14.6)	614 (14.3)	50 (22.3)	
Secondary section	542 (11.9)	502 (11.7)	40 (17.9)	
Operative spontaneous	166 (3.66)	157 (3.64)	9 (4.02)	
Admission to neonatal care (*n* = 4536)	909 (20.0)	805 (18.7)	104 (46.4)	*p* < 0.001
Hypoglycaemia (*n* = 4548)	133 (2.92)	86 (1.99)	47 (20.9)	*p* < 0.001
Respiratory distress (*n* = 4548)	228 (5.01)	213 (4.93)	15 (6.67)	*p* = 0.244
Male sex (*n* = 4548)	2381 (52.4)	2276 (52.7)	105 (46.7)	*p* = 0.077

Data are expressed as absolute numbers and percentages (in parentheses); ^a^*GDM* gestational diabetes mellitus, ^b^SGA – small for gestational age, ^c^*LGA* large for gestational age, ^d^two-tailed χ2 test

Mothers’ GDM also influenced gestational age at birth and the mode of delivery (Table 3). Neonates of women with diabetes were more often delivered prematurely at 32–36 weeks gestational age (11.1% versus 6.96%, *p* = 0.031). Compared to pregnant women without diabetes, those with GDM delivered their babies more often by primary or secondary C-sections (40.2% versus 26.0%, *p* < 0.001). The newborns of women with diabetes were more frequently admitted to the neonatal care unit (46.4% versus 18.7%, *p* < 0.001) and had a higher incidence of neonatal hypoglycaemia (20.9% versus 1.99%, p < 0.001) than newborns of mothers without diabetes.

Using a multiple logistic regression model, we evaluated the association between GDM and birth outcomes (Table 4). GDM doubled the relative risk ratio for primary and secondary C-sections (RRR 1.76, 95% CI 1.21–2.56, p < 0.001, and RRR 2.00, 95% CI 1.35–2.97, p < 0.001, respectively). Expecting mothers with GDM received more frequent positive results on the vaginal swab test than those without diabetes (OR 2.01, 95% CI 1.41–2.88, p < 0.001). Women with GDM delivered their babies 0.77 weeks (p < 0.001) earlier than women without GDM.

**Table 4:** Risk factors and their effect on pregnancy outcomes, with gestational diabetes as the independent variable

	Odds ratio (95% CI^a^)	*p*-value^d^
Vaginal infections (*n* = 3297)	2.01 (1.41–2.88)	*p* < 0.001
Macrosomia (*n* = 3934)	2.43 (1.41–4.18)	*p* < 0.001
Hypoglycaemia (*n* = 3934)	11.71 (7.49–18.30)	*p* < 0.001
Admissions to neonatal care (*n* = 3924)	4.18 (3.09–5.65)	*p* < 0.001
Respiratory distress (*n* = 3934)	1.59 (0.89–2.81)	*p* = 0.114
Relative risk ratio (95% CI)		
LGA^b^ (*n* = 3934)	1.71 (1.17–2.50)	*p* = 0.05
SGA^c^ (*n* = 3934)	0.64 (0.33–1.24)	*p* = 0.189
Mode of delivery (*n* = 3923)		
Spontaneous	Reference	
Primary section	1.76 (1.21–2.56)	*p* = 0.003
Secondary section	2.00 (1.35–2.97)	*p* = 0.001
Operative spontaneous	1.21 (0.55–2.67)	*p* = 0.642
ß (95% CI)		
Gestational age (*n* = 3928)^e^	−0.78 (− 1.09 – − 0.48))	*p* < 0.001

^a^*CI* confidence interval, ^b^*LGA* large for gestational age, ^c^*SGA* small for gestational age; ^d^linear regression (continuous outcomes), logistic regression (dichotomous outcomes) or multinomial logistic regression (categorical outcomes) adjusted for mother’s age, alcohol use and prepregnancy BMI; ^e^difference in the mean in weeks of gestational age at delivery

Also neonates were exposed to adverse effects of GDM (Table 4). The newborns of women with GDM were at an increased risk of being macrosomic (OR 2.43, 95% CI 1.41–4.18, p < 0.001), suffering from hypoglycaemia (OR 11.71, 95% CI 7.49–18.30, p < 0.001), and being admitted more often to neonatal care (OR 4.18, 95% CI 3.09–5.65, p < 0.001).

We did not observe that GDM influenced the prevalence of intrauterine death, neonatal resuscitation in the delivery room, congenital malformations, respiratory distress syndrome (ICD codes P22.0, P22.1, P22.8, and P22.9), premature labour, or premature rupture of membranes (data not shown).

## Discussion

The cumulative incidence of GDM reported in this paper is much higher than that in official perinatal statistics reported for Germany [1, 2] but is similar to the estimated prevalence of GDM published by Huy et al. from the German KiGGS study [30]. Other studies, including the Generation R study [14], reported a prevalence ratio of up to 22%. The main factors affecting these results are not only the ethnicity, age and BMI of pregnant women but also the lack of comprehensive screening for gestational diabetes, which would be offered to all women. The importance of such a general screening test has recently been demonstrated by Melchior et al. [6]. According to the authors, who analysed the billing data of health insurance companies, the prevalence of GDM in Germany in the first year after the introduction of a general screening test for GDM was above 13% in 2014 and 2015. Therefore, this high number of diagnosed GDM cases arises due to not only increased adipose tissue in pregnant women and women of older maternal age but also better diagnostic approaches.

### Predominant mediators of increased risk of GDM

Among many possible mediators of the increased risk of GDM, maternal age and prepregnancy BMI are the predominant factors. Our analyses showed that the risk of GDM increased by approximately 6% for each year of age. This increase in the risk is in line with findings reported in other publications [6, 31–33], independently of other risk factors.

Prepregnancy BMI was the second predominant mediator of the increased risk of GDM. Overweight and obese women were at higher risk of developing GDM, independently of other factors, such as maternal age, educational status, smoking or alcohol abuse. Since higher BMI values are one of the main risk factors in type 2 diabetes mellitus, it is no wonder that a similar correlation is observed between GDM and BMI. Comparable associations have been published elsewhere [14, 34, 35]. Moreover, a long-term follow-up study has demonstrated that the treatment of existing GDM is not sufficient to reduce childhood obesity [34]; therefore, a preconceptual approach is necessary [35].

### Socioeconomic status and gestational diabetes

Several previous studies have reported on the correlation of educational level [14, 15, 36–38], which is often used as a measure of socioeconomic status, with adverse progress and/or outcomes of pregnancy, including an increased risk of GDM. Considering the relation between socioeconomic status and GDM, different authors speculate that persons with lower education and/or income levels have worse health and/or dietary status, leading to overweight and obesity, which are preconditions of GDM [14, 15, 30, 38]. Our analyses showed a significant correlation between available income and risk of GDM, confirming that the previous hypothesis related to socioeconomic status is also valid for rural cohorts, such as the cohort of the SNiP. Even if our data from the regression analysis seem to confirm the hypothesis that low income is a potential risk factor for GDM, any direct comparison between individual studies is difficult, as there is no common definition of socioeconomic status.

The results published by Bouthoom et al. [14], based on the data from the Generation R cohort study from Rotterdam, showed a clear link between the educational levels of pregnant women and an increased risk of GDM. The group with the lowest educational level had twice the risk of GDM as the group with university-level education. A similar relationship was observed in our study, indicating a more general nature of the relationship, which is apparently independent of the ethical composition of the studied cohort.

Some published studies, such as the German KiGGS-Study [30], used a composite index combining educational level and professional attainment. Although one can view this approach as more general, the combination of available income and achieved educational level may suffer from systemic bias. For example, students having low income and a high educational level may receive similar social status to a person having low educational status and very high income. This phenomenon is visible in the reported data, which show that housewives, students/trainees, uneducated persons and educated women with intermediate professional qualifications had a similar risk of GDM to women with greater qualifications and/or in a management role [30].

### Alcohol consumption and gestational diabetes

It is suggested that alcohol consumption is associated with type 2 diabetes in a U-shaped fashion [39, 40]. Low to moderate alcohol consumption may have a protective effect against the development of type 2 diabetes. This finding might also explain the observed protective effect of alcohol against the incidence of GDM in our study. This effect has also been observed by Bouthoom and colleagues in the Generation R study in the Netherlands [14]. However, alcohol is a highly neurotoxic and teratogenic substance not only to the women consuming alcohol but also to the unborn child. Therefore, the general advice is to stop drinking alcohol as soon as the pregnancy is known.

### GDM and birth outcome

The link between a mother’s GDM and negative outcomes for the newborn infant and for the mother are well established and broadly accepted [41–43]. Therefore, it is not surprising that neonates born to women with diabetes were much heavier, were more often born prematurely and were more often delivered by C-section than children of mothers without diabetes. In general, the higher number of premature deliveries and C-sections among women with GDM can be explained by faster intrauterine growth due to overexposure to the energy source.

Neonatal hypoglycaemia is one of the most frequent adverse effects of exposure to GDM. Children suffering from neonatal hypoglycaemia may develop motor impairments and learning and behavioural difficulties [44–46]. There is an established and accepted relationship between neonatal hypoglycaemia and GDM, which, in turn, is facilitated by mothers’ high BMI values [47, 48].

The prevalence of the neonatal hypoglycaemia depends on nutritional status, gestational age and onset of feeding. Approximately 2 to 4% of mature newborns are affected, compared to 5 to 10% of premature babies and up to 50% of babies in GDM pregnancies [48]. Comparing these figures with the data of our study, we observe a much lower incidence of hypoglycaemia in neonates born to GDM mothers. These findings may be an indication for the appropriate therapy applied to this group of expecting mothers. However, the metabolome and epigenome of the offspring are affected by maternal BMI and glycaemia, suggesting long-term consequences for the next generation [49, 50].

### Strengths and limitations of the study

The SNiP covered approximately 95% of newborns and included almost 75% of all deliveries in the study area [23]. The collected data are, therefore, population-based and describe in detail the population of newborns and their mothers in eastern Pomerania between 2002 and 2008. The population-based design, the ethnic homogeneity, the large number of participants enrolled, the vast amount of information collected and the area covered create a unique selling point for the SNiP [23]. When compared to relevant national or international studies on child health, such as Generation R from Rotterdam [14], LIFE Child from Leipzig [19] or Ulmer SPATZ from Ulm [51], which are located in large cities, the SNiP cohort comes from a rural area that is affected by intensive agriculture but lacks large industry.

In this paper, we show only restricted analysis of alcohol and tobacco consumption. This may be considered to be a limitation of our analysis. However, current literature shows the feasibility of this methodology. In the LAMBS study [52], the adverse effects of tobacco and alcohol consumption on late and moderate preterm birth (LMPB) were investigated. Similar to us, the authors restricted their analysis to the time point, at which consumption ceased, without considering the quantified tobacco and alcohol consumption. Also, Pfinder and colleagues [53], who analysed factors associated with preterm births and SGA in two large Western-European studies, KiGGS and ABCD, did not consider the smoking pattern during pregnancy.

A limitation of the study is that there was a screening for GDM by only qualitative glucose measurement in urine. A glucose tolerance test was only conducted in cases of positive urine measurement. This procedure might result in underestimation of the incidence of GDM in our cohort. However, we have shown that the prevalence of GDM in the study region is almost twice as high as the prevalence officially reported for Germany.

## Conclusions

We have shown that the risk of developing GDM increases with women’s age and prepregnancy BMI. We demonstrated that GDM results in serious negative outcomes at birth for mothers and their offspring, with possible long-term effects on their health. As the risk of GDM increases with mothers’ BMI, age, and low-income status, those factors should be taken into account when preventive intervention strategies are developed and the target risk group is established. The high incidence of GDM reported in this paper is clear evidence of the need for general screening for GDM.

## Abbreviations

BMI: Body mass index
CI: Confidence interval
GDM: Gestational diabetes mellitus
GWG: Gestational weight gain
LGA: Large for gestational age
n: Number of cases included in a particular analysis
oGTT: Oral glucose tolerance test
OR: Odds ratio
RRR: Relative risk ratio
SEP: Socioeconomic position
SGA: Small for gestational age
SNiP: Survey of Neonates in Pomerania
